# Supplementary material for: Likelihoods for a general class of ARGs under the SMC
Source: Genetics. 2025 May 29;232(1):iyaf103. doi: 10.1093/genetics/iyaf103 (PMC12774825; doi:10.1093/genetics/iyaf103)
Supplement: iyaf103_Supplementary_Data [file iyaf103_supplementary_data.pdf]

Algorithm 1: LogDepth: pseudocode for equation Eq. (4)

Input: Count vector C; interval boundaries T;
Parent time t_p; child time t_c;
Initial index i_0; recombination rate r;
Coalescence rate c; recombination flag R
Output: log likelihood contribution in time dimension

Initialize cumulative area $A \leftarrow 0$;
Initialize result $S \leftarrow 0$;
Set index $i \leftarrow i_0$;
Define helper function $f(f_0, f_1)$:

- If $f_0 = f_1$, return 0
- Else return $\dfrac{c}{r - cf_1} \cdot \dfrac{c}{r - cf_0} \cdot (f_1 - f_0) \cdot \dfrac{r}{c}$

$t_{\text{stop}} \leftarrow t_p$
if $R = \mathit{False}$ **then**
  $t_{\text{stop}} \leftarrow t_c$
while $T_i > t_{stop}$ **do**
  $A \leftarrow A + (T_i - T_{i-1}) \cdot C_{i-1}$
  $i \leftarrow i - 1$
  if $i < 1$ **then**
    break
if $R = \mathit{False}$ **then**
  return $-c \cdot A$
$B \leftarrow -r/(r - cC_{i-1}) \cdot \exp(-rT_i - cA)$
while $i > 0$ **do**
  $A \leftarrow A + (T_i - T_{i-1}) \cdot C_{i-1}$
  $i \leftarrow i - 1$
  if $T_i = t_c$ **then**
    break
  $S \leftarrow S + f(C_{i-1}, C_i) \cdot \exp(-rT_i - cA)$
$S \leftarrow B + S + r(r - cC_i) \cdot \exp(-rT_i - cA)$
return $\log(S)$

Algorithm 2: LogLikelihood: pseudocode for Eq. (5)

Input: Time vector I for all nodes;
Edge arrays containing parent, child, left and right coordinate of each edge: `edges_parent`, `edges_child`, `edges_left`, `edges_right`;
Recombination rate r, coalescence rate c;
`treesequence`;
Output: Total log-likelihood value

Initialize log-likelihood: LL $\leftarrow 0$;
Initialize lineage count vector $C \leftarrow 0$;
Initialize arrays to track last parent and visited nodes ;
while *next tree exists in treesequence* **do**
    `// Process edge removals`
    foreach *edge e being removed* **do**
        Identify parent p and child c from e ;
        Get node times t_p for p, and t_c for c ;
        Decrease lineage count C between t_c and t_p ;
        Record p as last parent of c ;
    `// Process edge insertions`
    foreach *edge e being inserted* **do**
        Identify parent p and child c from e ;
        Get node times t_p for p, and t_c for c ;
        Set `rec_event` $\leftarrow$ False ;
        Set `left_parent_time` $\leftarrow \infty$;
        if *child c had a previous parent* p' **then**
            Retrieve previous parent time $t_{p'}$;
            if $p' \neq p$ *and p not visited* **then**
                Set `rec_event` $\leftarrow$ True ;
        Set $t_{p_{\min}} \leftarrow \min(t_p, \texttt{left\_parent\_time})$ as effective parent time ;
        `// Add depth contribution`
        LL $\leftarrow$ LL + LogDepth($C, I, t_{p_{\min}}, t_c, p, r, c,$ `rec_event`)
        `// Add span contribution`
        LL $\leftarrow$ LL $- r \cdot (t_p - t_c) \cdot (\texttt{edges\_right}[e] - \texttt{edges\_left}[e])$
        `// Update lineage count`
        Increase C between t_c and t_p ;
Number of nodes with out-degree 0: n_o ;
Contribution coalescent events: $ll_c = \text{num\_edges} - \text{num\_nodes} + n_o$;
Number of nodes with in-degree 0: n_r ;
Contribution recombination events: $ll_r = \text{num\_edges} - \text{num\_nodes} + n_r$;
Update likelihood: LL $\leftarrow$ LL $+ ll_c \cdot \log(c) + ll_r \cdot \log(r)$;
return LL

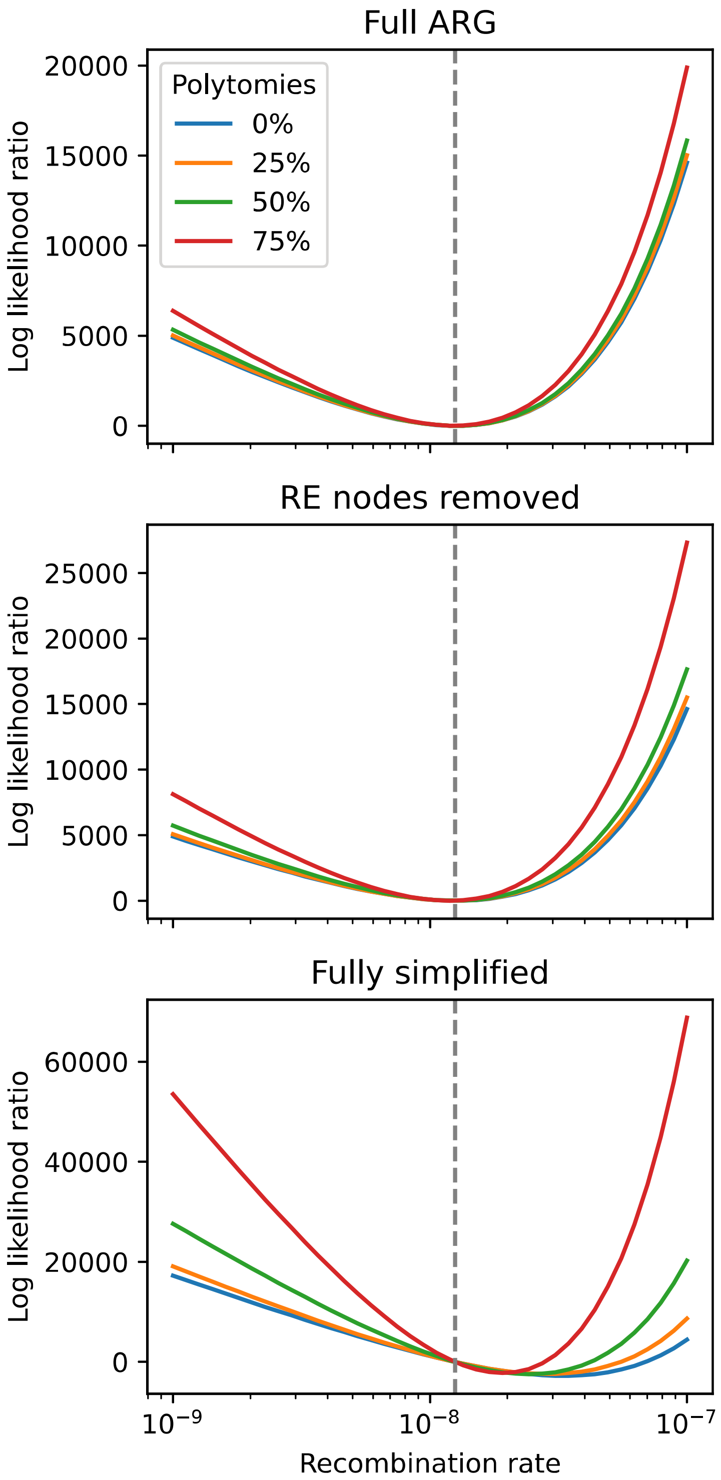

Figure S1: Log likelihood-ratio curves for three different ARGs with increasing fractions of polytomies. The base ARGs are as described in Fig. 3, but here we remove 25%, 50% and 75% of the internal nodes to create polytomies. The true value of the recombination parameter is indicated by the dashed line, as in Fig. 3.
